# Risk of depression and self-harm in teenagers identifying with goth subculture: a longitudinal cohort study

**DOI:** 10.1016/S2215-0366(15)00164-9

**Published:** 2015-08-27

**Authors:** Lucy Bowes, Rebecca Carnegie, Rebecca Pearson, Becky Mars, Lucy Biddle, Barbara Maughan, Glyn Lewis, Charles Fernyhough, Jon Heron

**Affiliations:** a Department of Experimental Psychology, University of Oxford, Oxford, UK; b Centre for Academic Mental Health, Addiction and Suicide Research, School of Social & Community Medicine, University of Bristol, Bristol, UK; c MRC Social, Genetic and Developmental Psychiatry Research Centre, Institute of Psychiatry, Psychology and Neuroscience, King's College London, London, UK; d Division of Psychiatry, Faculty of Brain Sciences, University College London, London, UK; e Developmental Psychology, Durham University, Durham, UK

## Abstract

**Background:**

Previous research has suggested that deliberate self-harm is associated with contemporary goth subculture in young people; however, whether this association is confounded by characteristics of young people, their families, and their circumstances is unclear. We aimed to test whether self-identification as a goth is prospectively associated with emergence of clinical depression and self-harm in early adulthood.

**Methods:**

We used data from the Avon Longitudinal Study of Parents and Children, a UK community-based birth cohort of 14 541 pregnant women with expected delivery between April 1, 1991, and Dec 31, 1992. All children in the study were invited to attend yearly follow-up visits at the research clinic from age 7 years. At 15 years of age, participants reported the extent to which they self-identified as a goth. We assessed depressive mood and self-harm at 15 years with the Development and Wellbeing Assessment (DAWBA) questionnaire, and depression and self-harm at 18 years using the Clinical Interview Schedule-Revised. We calculated the prospective association between goth identification at 15 years and depression and self-harm at 18 years using logistic regression analyses.

**Findings:**

Of 5357 participants who had data available for goth self-identification, 3694 individuals also had data for depression and self-harm outcomes at 18 years. 105 (6%) of 1841 adolescents who did not self-identify as goths met criteria for depression compared with 28 (18%) of 154 who identified as goths very much; for self-harm, the figures were 189 (10%) of 1841 versus 57 (37%) of 154. We noted a dose–response association with goth self-identification both for depression and for self-harm. Compared with young people who did not identify as a goth, those who somewhat identified as being a goth were 1·6 times more likely (unadjusted odds ratio [OR] 1·63, 95% CI 1·14–2·34, p<0·001), and those who very much identified as being a goth were more than three times more likely (unadjusted OR 3·67, 2·33–4·79, p<0·001) to have scores in the clinical range for depression at 18 years; findings were similar for self-harm. Associations were not attenuated after adjustment for a range of individual, family, and social confounders.

**Interpretation:**

Our findings suggest that young people identifying with goth subculture might be at an increased risk for depression and self-harm. Although our results suggest that some peer contagion operates within the goth community, our observational findings cannot be used to claim that becoming a goth increases risk of self-harm or depression. Working with young people in the goth community to identify those at increased risk of depression and self-harm and provide support might be effective.

**Funding:**

Wellcome Trust, Medical Research Council Programme.

## Introduction

Depression is the leading contributor to the worldwide burden of disease in young people aged 10–24 years.^1^ Rates of depression increase substantially during adolescence,^2^, ^3^ a period of transition characterised by social, emotional, and physiological changes. Understanding of specific risk factors during this important transition is therefore needed to inform prevention strategies. During this period, peers are the main sources for social comparison and appraisal, and self-consciousness is heightened.^4^ Studies have shown that peers report similar levels of depressive symptoms to each other,^5^ although evidence for peer contagion effects on depression and related phenotypes (eg, self-harm) is mixed.^6^, ^7^, ^8^, ^9^, ^10^ Identification of young people at heightened risk of depression and related phenotypes is a key area for future research.^10^ A strong and robust increased risk of self-harm and attempted suicide has been reported in young people identifying with contemporary goth subculture and related subcultures generally described as alternative youth.^11^ A goth is defined in the Oxford Dictionary as “a member of a subculture favouring black clothing, white and black make-up, and goth music”. Much diversity exists within the goth subculture, making definition of the average adolescent goth difficult;^12^ however, many social norms are associated with being a goth, including alternative clothing and music, and a dark, morbid mood and aesthetic. The goth subculture has been suggested to provide an important source of validation and community to individuals who do not conform with societal norms.^12^

Why affiliation with goth subculture is associated with an increased risk of self-harm and whether it is also associated with increased depression is unclear. If the observed association represents underlying social transmission, such as emulation of subcultural icons or self-harming peers, we might postulate that the association would be specific to self-harm. A contrasting explanation is that the observed association represents shared exposure to stressors, or social selection mechanisms, whereby vulnerable young people are attracted to others with similar underlying risks. In this case, goth affiliation would be associated with related phenotypes (specifically depression) in addition to self-harm and suicidal ideation, and observed associations would be confounded by baseline characteristics of young people before identification as a goth, and concurrent risk factors that might be specific to this group (eg, peer victimisation). Notably, the study that identified an association between goth subculture identification and deliberate self-harm by Young and colleagues^11^ did not adjust for several salient early risk factors that could have confounded the association, including specific emotional and behavioural problems, peer victimisation, and maltreatment. With these issues in mind, we aimed to test whether self-identification with the goth subculture at 15 years of age was associated with self-harm and depression at 18 years of age in a large cohort of young people followed up prospectively.

**Research in context**

**Evidence before this study**

We searched PubMed to identify potential literature published before Nov 5, 2014, using the search terms “goth or emo or subculture” and “depress*, suic*, self-harm, or mental health”. We identified 98 articles, of which 2 examined an association between goth or “alternative youth” affiliation and depression or self-harm, only one of which was prospective in design.

**Added value of this study**

To our knowledge, our study is the largest so far to prospectively examine the association between self-identification with the goth subculture and later self-harm and depression. Our study was designed to address some of the limitations in the original study by adjusting for the potential confounding effects of specific emotional and behavioural problems, peer victimisation, and maltreatment.

**Implications of all the available evidence**

A strong, dose–response association between identification as “alternative” or “goth” and self-harm has now been reported in samples from Scotland, Germany, and England. Our study also identified a strong association with self-identification as a goth and adult depression. Together, these findings suggest that youths who identify with the goth subculture might represent a vulnerable group, although establishing causal links from these observational studies is not possible.

## Methods

### Study design and participants

The Avon Longitudinal Study of Parents and Children (ALSPAC) is a longitudinal cohort study that recruited pregnant women resident in the former county of Avon, UK, who had expected dates of delivery between April 1, 1991, and Dec 31, 1992 (the administrative county of Avon was abolished in 1996). 14 541 pregnant women initially enrolled and returned at least one questionnaire or attended a Children in Focus clinic. Of these 14 541 pregnancies, 68 have no known birth outcome; of the remaining 14 472 pregnancies, 195 were twins, three were triplets, and one was quadruplets, resulting in 14 676 known fetuses. The 14 541 pregnancies resulted in 14 062 live born children, of whom 13 988 were alive at 1 year of age (74 infants died). When the oldest children were about 7 years of age, an attempt was made to bolster the initial sample with eligible participants who had declined to join the study originally; a further 456 children were enrolled at this point. As a result, when taking into account the variables obtained from the age of 7 years onwards (and potentially abstracted from obstetric notes), data were available for more than the 14 541 pregnancies mentioned. The phases of enrolment are described in detail in the cohort profile report.^13^ A fully searchable data dictionary is available online .

Children were invited to attend annual assessment clinics, including face-to-face interviews and psychological and physical tests from the age of 7 years onwards. In this analysis, we included children who were still participating in the study and who completed a computer-based survey asking them about their self-identification with eight different social groups during clinic visits at 15 years of age.

Ethical approval for the study was obtained from the ALSPAC Ethics and Law Committee and the Local Research Ethics Committees. From the beginning of the study, ALSPAC has had its own Ethics and Law Committee. Initial ethical approval was obtained for gaining written, informed consent from pregnant mothers. At each follow-up clinic assessment, mothers provided informed written consent, and children provided assent after receiving a full explanation of the study. Written informed consent was obtained from participants for the clinic at 18 years.

### Procedures

We adapted the Peer Crowd Questionnaire^14^, ^15^ on the basis of interviews with focus groups of 14-year-old adolescents (seven girls, four boys) from a large comprehensive school in south Bristol, in the former county of Avon in January, 2006, to identify salient youth subcultures at that time and location. We identified eight different social groups: “sporty”, “populars”, “skaters”, “chavs”, “loners”, “keeners”, “bimbos”, and “goths”.

At 15 years of age, study participants were invited to attend the research clinic for the annual study follow-up visit. During the clinic, participants completed a computer-based survey in which they were asked a series of questions about their self-identification with these social groups. For self-identification as a goth, participants were asked, “is there a group of teens in your school or neighbourhood with the reputation of…rebelling against the norm (in clothing or ideas, for example), or in attempting not to conform to social ideals (eg, the ‘goths’)? How much do you identify with...the goths?” Participants responded “not at all”, “not very much”, “somewhat”, “more than somewhat”, or “very much”.

### Outcomes

The outcomes of the study were depression and self-harm at 18 years of age. We measured the prospective associations between self-identification with the goth subculture at 15 years of age and depression and self-harm at 18 years of age.

At the 15 year visit, we assessed depressive mood with the Development and Wellbeing Assessment (DAWBA).^16^ To account for the full range of symptoms of depression at the age of 15 years, we derived a total symptom count, summing all of the symptoms from the depression section of the DAWBA.

At the 18 year visit, participants completed a self-administered computerised version of the Clinical Interview Schedule-Revised (CIS-R)^17^ to assess depression. CIS-R is designed for, and has been widely used within, community samples.^18^ We used a binary variable (depressed or not depressed) to record depression; cases were those meeting the criteria for a primary diagnosis of mild, moderate, or severe depression with the CIS-R, which generates these diagnoses with algorithms based on the International Classification of Diseases (ICD)-10 criteria.

We assessed self-harm with self-report at the 15 year and 18 year research clinics. Self-harm was assessed at the 15 year research clinic with an item from the DAWBA: “over the whole of your lifetime have you ever tried to harm or hurt yourself?” At the 18 year clinic, we assessed self-harm with CIS-R; participants were classified as having a lifetime history of self-harm if they responded positively to the question “have you ever hurt yourself on purpose in any way (eg, by taking an overdose of pills or by cutting yourself)?” We did not distinguish between individuals who had harmed themselves with and without suicidal intent in the present study.

We assessed parental occupational social class on the basis of the lower of the mother's or mother's partner's occupational social class,^19^ dichotomised into professional, managerial, or skilled professions versus partly or unskilled occupations. We coded highest maternal education as the percentage of mothers with a university degree versus those without a degree. Both of these variables were measured during pregnancy. Maternal depression (assessed during pregnancy, at 18 weeks' gestation) was measured using a postal questionnaire based on the Edinburgh Postnatal Depression Scale^20^ (original internal consistency, a=0·87; in the present study, a=0·85). Maternal history of severe depression (self-report of past history of severe depression, yes or no) was also assessed by postal questionnaire at 12 weeks' gestation.

Peer victimisation was assessed when children were 8 and 10 years of age using a modified version of the Bullying and Friendship Interview Schedule.^21^ Children's exposure to life events at 3·5 years was assessed using maternal report. The items included in this questionnaire were taken from other studies.^22^, ^23^ Children's internalising and externalising problems were assessed using maternal reports from the Strengths and Difficulties Questionnaire (SDQ)^24^ when children were 11 years of age. Previous depression, defined as scores reaching clinical significance (scores of 11 or more on the Short Moods and Feelings Questionnaire, yes *vs* no), was assessed via self-report when children were 10, 13, and 16 years of age.^25^ This scale has been validated against the CIS-R.^26^ Any axis-1 disorders classified using the Diagnostic and Statistical Manual of Mental Disorders (DSM)-IV (no *vs* yes) were assessed using maternal reports from the DAWBA^27^ when children were 10 years old. Diagnoses were derived with a computer algorithm, which has been shown to be similar in accuracy to a clinical rating method.^16^

Children's self-perception was self-reported using a shortened form of Harter's Self Perception Profile for Children^28^ during a clinic when children were on average aged 8·5 years. This measure consisted of 12 items relating to worldwide self-worth and scholastic competence. We gave the Emotionality, Activity, and Sociability Temperament Scale^29^ questionnaire to mothers when children were aged 6 years old and this scale had four subscales (emotionality, shyness, sociability, and activity).

### Statistical analysis

We examined the univariable prospective association between identification with goth subculture and depression in logistic regression analyses, then adjusted for baseline depression at 15 years (using the continuous score derived from the DAWBA), and baseline self-harm at 15 years to minimise the possibility of reverse causality. We then additionally adjusted for a range of individual, family, and social confounders, including previous depression, internalising and externalising difficulties at 11 years, self-perception, victimisation by bullies, antenatal depression, maternal history of depression, maternal education, and temperament (emotionality and activity).

We selected covariates a priori from the scientific literature on the basis of their potential to confound the association between self-identification as a goth, depression, and self-harm.^3^, ^12^, ^30^ When data were subject to particularly high attrition, we used the earliest measure for the covariate, to maximise our sample size. We adjusted for depression at 15 years of age, and for many of the same confounding variables as the original study by Young and colleagues^11^ and a range of further confounding variables, including baseline self-harm, early emotional and behavioural difficulties, psychiatric disorder, peer problems, and child maltreatment.

Similarly to most prospective studies, missing data because of attrition were a concern. We used a sample with complete data across all exposure, outcome, and confounding variables (n=2351) to investigate main and independent effects of self-identification with the goth subculture. To address the possibility of bias, we used collected data to predict and impute missing variables and did analyses with imputed datasets, allowing individuals with incomplete data to be included in the analyses. We used a fully conditional specification as implemented in the Multiple Imputation by Chained Equation algorithm in Stata version 12.^31^, ^32^

The ALSPAC sample has substantial information on sociodemographic variables that predict missing data, allowing the construction of an imputation model using strong auxiliary information. Because missing data in the ALSPAC study have been previously found to be dependent on several variables (eg, gender, parental social, and maternal education), these variables were included in the imputation models, in addition to other measures that have been identified as closely associated with adolescent self-harm and with our outcome variables (eg, similar measures from the domains of mental health or substance use obtained earlier in the study) and all other variables included in analyses (appendix). Variable estimates were averaged from 100 imputed datasets using Rubin's rules.^32^ In longitudinal studies, earlier measures of child depression can be used to predict later depression,^33^ allowing us to impute up to a starting sample of 5342, those with at least one measure of adolescent depression and complete exposure data. The imputations were separated into these two stages to establish that extending the model to those without any earlier depression data produced similar results. Analyses were done using Stata version 12 (StataCorp, TX, USA). We deemed p values less than 0·05 as significant.

### Role of the funding source

The funders of the study had no role in study design, data collection, data analysis, data interpretation, or writing of the report. LBo, RC, RP, and JH had full access to all of the data (including statistical reports and tables) in the study and can take responsibility for the integrity of the data and the accuracy of the data analysis. The corresponding author had full access to all the data in the study and had final responsibility for the decision to submit for publication.

## Results

**Figure 1:**
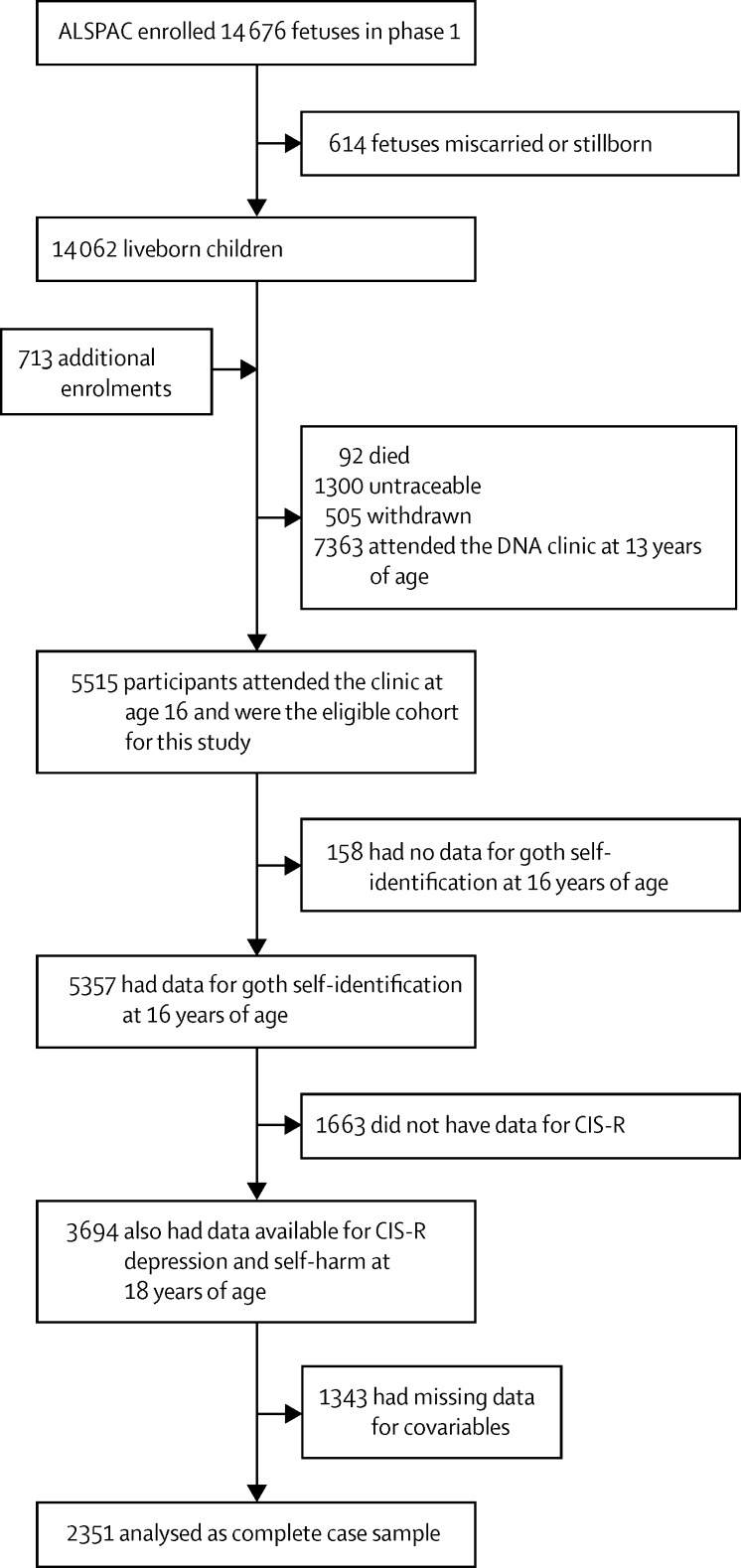
Flow chart of ALSPAC study participants ALSPAC=The Avon Longitudinal Study of Parents and Children. CIS-R=Clinical Interview Schedule-Revised.

10 962 adolescents in the ALSPAC study were invited to attend the 15 year clinic visit, of whom 5515 attended (average age of 15·5 years [SD 0·4]) and 5357 completed the survey about subculture identification. From the eight subcultures identified, the goth subculture was recognised by all adolescents interviewed. The most frequently endorsed subcultures were “sporty”, “populars”, “skaters”, “chavs”, and “goths”. At the 18 year visit, 3694 adolescents attended and provided outcome data (mean age 17·8 years [SD 0·5]). Overall, subculture identification and outcome data at 18 years were available for 3694 adolescents (figure 1).

Participants lost to follow-up between the 15 and 18 year visits were no more likely to self-identify as a goth (χ^2^ 0·558, p=0·455), to have self-harmed by the age of 15 years (χ^2^ 2·001, p=0·157), or to have shown high levels of depressive symptoms at 15 years of age (χ^2^ 9·713, p=0·205) than were those with data for all variables. Table 1 shows how individuals who identified as goths “more than somewhat” to “very much” differed in early individual and family characteristics (assessed between the age of 8 years and 15 years) compared with young people who reported they “did not” or “somewhat” self-identify with the goth subculture. Those who identified with the goth subculture were more likely to be girls. They were also more likely to have mothers with a history of depression, report being bullied at the age of 8 and 10 years, and have a history of emotional and behavioural difficulties, according to mother's reports from the SDQ, including symptoms of depression and anxiety, hyperactivity, and peer relationship difficulties. Young people who self-identified with the goth subculture also self-reported more symptoms of depression at 10, 12, and 13 years of age. However, generally, the magnitude of these differences was small and unlikely to be clinically significant for most participants; p values should be interpreted with caution in view of the large sample and number of comparisons made.

**Figure 2:**
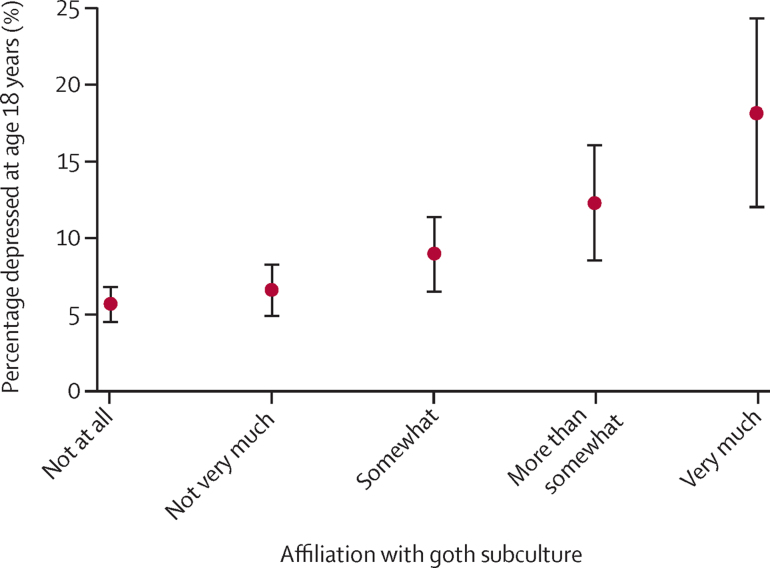
Percentage of sample depressed according to affiliation with goth subculture based on the trend in table 2

In our sample of young people with complete data for goth self-identification and depression at 18 years, 105 (6%) of the 1841 individuals who responded that they did not at all identify with the goth subculture had depression scores in the clinical range at 18 years compared with 47 (9%) of the 523 young people who identified with goth subculture “somewhat” and 28 (18%) of the 154 individuals who responded that they identified with the goth subculture “very much” (figure 2).

Table 2 shows clear dose–response associations between the extent to which young people self-identified as a goth and depression scores in the clinical range at 18 years. For example, compared with young people who did not identify as a goth, those who somewhat identified as being a goth were 1·6 times more likely to have scores in the clinical range for depression at 18 years (unadjusted odds ratio [OR] 1·63, 95% CI 1·14–2·34, p<0·001) and were more than three times as likely to have scores in the clinical range for depression at 18 years (unadjusted OR 3·67, 2·33–4·79, p<0·001). For each unit increase in goth affiliation, the (unadjusted) odds of depression increased by 1·36 (1·23–1·49). Adjustment for potential confounders led to only a fairly small attenuation of this OR in the final model (OR 1·27, 1·11–1·47; table 3). As expected, we noted the largest attenuation after adjustment for baseline symptoms of depression (OR 1·29, 1·15–1·45).

We also identified evidence of a dose–response association between goth identification and self-harm at 18 years of age. For example, compared with young people who did not identify as a goth, those who indicated that they somewhat identified as being a goth were 2·33 times more likely to report having self-harmed, whereas those who very much identified themselves as being a goth were more than five times more likely to report self-harm. For each unit increase in affiliation with goth subculture, the (unadjusted) OR of self-harm increased by 1·52 (1·42–1·63) The OR decreased by 12·5% in the adjusted model (OR 1·33, 1·19–1·48; table 3).

Of all subcultures identified, 154 young people who identified with the goth subculture very much were most at risk of depression and self-harm, with 28 (18%) having scores in the clinical range for depression, and 57 (37%) reporting self-harm at 18 years of age. Of the 341 young people who identified as skaters very much, 37 (11%) had depression, and 85 (25%) reported self-harm at 18 years of age; and of the 47 adolescents who self-identified as loners, 4 (9%) had depression scores in the clinical range, and 12 (26%) reported self-harm. Those who self-identified as sporty were least likely to have depression and to have self-harmed at age 18 years (31 [4%] of 786 sporty individuals had depression and 47 [6%] had self-harmed).

## Discussion

In this analysis of data from a longitudinal cohort study, we noted a dose–response association between the extent to which young people self-identified with the goth subculture at the age of 15 years and both depression and self-harm at 18 years of age. This association was independent of the potentially confounding characteristics of previous depressive symptoms and self-harm, personality, history of bullying, behavioural issues, maternal depression, and perception of body image.

Young people who self-identified as goths were more likely to be girls (contrasting with the findings from the original Young and colleagues' study sample in Glasgow in which they were more likely to be boys^11^), to have mothers with a history of depression, to have a history of emotional issues, including depression themselves, and to report issues with peers, including being bullied. Such vulnerability factors for depression suggest a degree of self-selection, with young people more susceptible to depression and self-harm being more likely to be attracted to the goth subculture. Yet, even after adjustment for these early risk factors, young people who self-identified as goths remained at an increased risk of depression and self-harm compared with those who did not identify with the subculture. Although some residual confounding is likely to remain, our findings support earlier evidence^11^ that goths represent a vulnerable group.

Our study has several strengths, including the large sample size, the prospective design from before birth to 18 years (a time when rates of depression peak), and the detailed information on a range of potential confounding variables. Individuals who are susceptible to depression might be more drawn to subcultures, such as the goth subculture, which are known to embrace marginalised individuals from all backgrounds, including those with previous mental health difficulties. Thus, as we originally postulated, the reported association between goth affiliation and depression could be due to social selection factors not addressed adequately in the previous study.^11^ To investigate this, we adjusted the analysis for concurrent depressive symptoms and controlled for the effects of previous victimisation, emotional and behavioural issues, and a range of other potential confounders to test whether noted associations remained. Although we adjusted for many potential confounders, we cannot exclude the possibility of residual confounding. A second limitation is the loss to follow-up from the original ALSPAC sample. Young adults who attended the clinic at 18 years of age came from families with higher levels of education and social class, which might have reduced statistical power in detection of an association between goth affiliation at 15 years and depression and self-harm at 18 years. The wealth of data about participants who have not been followed up in ALSPAC makes the assumptions behind our handling of missing data much more reasonable, and the results of our imputation analyses were consistent with our complete case findings. We therefore think that such a strong association is unlikely to be explained by attrition. The questions used to assess self-harm at the age of 15 and 18 years were worded differently and could have led to different responses by participants. Our definition of self-harm included individuals who had self-harmed with and without suicidal intent. The extent to which suicidal and non-suicidal self-harm represent distinct concepts or more or less extreme versions of the same behaviour is a source of debate.^34^, ^35^, ^36^, ^37^, ^38^ Further research should examine whether associations with goth subculture and self-harm differ according to self-reported suicidal intent. Finally, our findings support those of Scottish^11^ and German^39^ young people, reporting a link between goth affiliation and self-harm; however, whether our findings generalise beyond these populations is unclear. The meaning and cultural identity of goths are likely to vary within and across cultures and time. Only eight subcultures were investigated and therefore the role of young people who identify with rarer youth subcultures is not known and is an important direction of future research.

Peer contagion might represent one mechanism by which young people who affiliate with other at-risk goths might be at increased risk of depression and self-harm. Evidence of peer contagion effects for both depression and self-harm have been previously reported.^40^ Although corumination, excessive reassurance seeking, and negative feedback seeking might represent mechanisms through which peer contagion increases risk of depression,^41^ why peer contagion might operate to increase risk of self-harm is unclear. Such effects might arise if young people actively discuss self-harming as an effective emotion regulation strategy. In a study by Young and colleagues,^39^ young people who identified with “alternative” subcultures (including goths) were more likely to report autonomic reasons for self-harming (ie, to reduce negative emotions), including to “stop bad feelings” and to “relieve feeling numb or empty” compared with non-alternative young people. Alternative young people were also more likely to endorse social reasons for self-harming (eg, to “feel more part of a group”). Emulation of peer behaviour has also been suggested,^40^ and in the context of the goth subculture, this might have some validity. Information about exposure to self-harm in others was not available in this study but is an important area for future research.

Well validated experimental manipulations that use sad music to induce symptoms of depression exist,^36^, ^37^ although effects in the laboratory are short lasting. Listening to repeated music from the goth genre might lower mood and exacerbate symptoms of depression. An alternative explanation for our findings could be an affiliation or attraction model in which the extent to which young people self-identify with the goth subculture might represent the extent to which at-risk young people feel isolated, ostracised, or stigmatised by society. Such young people might be attracted to other alternative goth young people who do not adhere to societal norms. The registering of hate crimes committed against goths and other subcultures by Manchester police after the murder of 20-year-old goth Sophie Lancaster in 2007 suggests that goths might be the target of social stigma and aggression.

Although our findings suggest that youths who identify with the goth subculture might represent a vulnerable group, our observational findings cannot be used to claim that becoming a goth causes an increased risk of self-harm and depression. Although peer contagion might operate within the goth youth community, other factors such as stigma and social ostracising might represent the underlying mechanisms of increased risk. Working with youths in the goth community to identify those at risk of depression and self-harm and provide support might be effective. Public campaigns to reduce stigma and aggression targeted to individuals from diverse subcultures might also be important.

## Figures and Tables

**Table 1:** Baseline characteristics of study participants according to goth self-identification

		**Low goth self-identification (n=4709)**	**High goth self-identification (n=648)**	**p value**
Gender				
	Female	2453 (52%)	374 (58%)	0·01
	Male	2256 (48%)	274 (42%)	0·01
Parental social class				
	High (professional, managerial, or skilled occupations)	2072 (44%)	286 (44%)	0·94
	Low (partly skilled or unskilled occupations)	2637 (56%)	362 (56%)	0·95
Mother with degree		871 (18%)	119 (18%)	0·94
Maternal depression score during pregnancy		6·5 (4·8)	7·0 (5·1)	0·02
Maternal history of severe depression		283 (6%)	64 (10%)	<0·001
Bullying				
	Bullied at age of 8 years	1822 (39%)	307 (47%)	<0·001
	Bullied at age of 13 years	542 (12%)	106 (16%)	0·02
Life events score at age 3·5 years		2·7 (2·5)	3·1 (2·8)	<0·001
Strengths and difficulties (age 12 years)				
	Prosocial score	8·4 (1·6)	8·3 (1·7)	0·13
	Hyperactivity score	2·6 (2·1)	2·8 (2·2)	0·04
	Emotional symptoms score	1·4 (1·7)	1·6 (1·7)	0·03
	Conduct problems score	1·1 (1·3)	1·1 (1·3)	0·24
	Peer problems score	1·0 (1·4)	1·3 (1·6)	<0·001
	Total difficulties score	5·8 (4·4)	6·5 (4·7)	<0·001
MFQ score >11				
	MFQ>11 at age of 10 years	198 (4%)	27 (4%)	0·97
	MFQ>11 at age 12·5 years	231 (5%)	51 (8%)	<0·001
	MFQ>11 at 13·5 years	364 (8%)	115 (18%)	<0·001
DAWBA depression score at age of 15 years		3·2 (4·5)	5·7 (6·7)	<0·001
Self-harm at age of 15 years		457 (10%)	166 (26%)	<0·001
Self-perception at age of 11 years				
	Kind	3899 (83%)	509 (79%)	<0·001
	Friendly	4309 (92%)	584 (90%)	0·02
	Confident	2731 (58%)	360 (56%)	<0·001
	Sporty	2816 (60%)	262 (40%)	<0·001
	Good looking	1681 (36%)	190 (29%)	<0·001
	Easily bored	1333 (28%)	226 (35%)	0·01
	Different from others	739 (16%)	222 (34%)	<0·001
	Worries a lot	782 (17%)	154 (24%)	<0·001
	Messes about a lot	1267 (27%)	211 (33%)	0·02
Temperament at age of 6 years (child in top quartile)				
	Emotionality	749 (16%)	157 (24%)	<0·001
	Activity	739 (16%)	80 (12%)	0·20
	Shyness	871 (18%)	120 (19%)	0·94
	Sociability	876 (19%)	123 (19%)	0·17

Data are n (%) or mean (SD). Self-identification by binary variable of low self-identification (not at all to somewhat) or high self-identification (more than somewhat to very much).

**Table 2:** Odds ratio for depression and self-harm at 18 years for each category of goth identification

	**Number of participants (n=5357)**	**Number of participants with data for goth self-identification at 15 years and outcomes at 18 years (n=3694)**	**Depression**		**Self-harm**	
			OR (95% CI)	p value	OR (95% CI)	p value
Linear effect*	5357	3694	1·36 (1·23–1·49)	p<0·001	1·52 (1·42–1·63)	p<0·001
Not at all	2759	1841	Reference	..	Reference	..
Not very much	1234	884	1·16 (0·83–1·62)	..	1·52 (1·20–1·93)	..
Somewhat	716	523	1·63 (1·14–2·34)	..	2·33 (1·80–3·02)	..
More than somewhat	410	292	2·33 (1·56–3·47)	..	3·65 (2·72–4·89)	..
Very much	238	154	3·67 (2·33–4·79)	..	5·14 (3·58–7·36)	..
LR test	5357	3694	χ^2^ 38·99	p<0·001	χ^2^ 132·65	p<0·001

OR=odds ratio. LR=likelihood ratio.

* Goth identification was treated as a continuous variable; therefore, the linear effect refers to OR of depression for a one point increase in goth identification. The LR test provides a test for the dose–response trend.

**Table 3:** Odds ratio for depression and self-harm given for each category increase in goth identification

	**Depression**		**Self-harm**	
	**OR (95% CI)**	**p value**	**OR (95% CI)**	**p value**
Unadjusted (n=3694)	1·36 (1·23–1·49)	p<0·001	1·52 (1·42–1·63)	p<0·001
Complete cases (n=2351)	1·39 (1·22–1·58)	p<0·001	1·51 (1·37–1·66)	p<0·001
Adjusted for baseline depression score (n=2351)	1·33 (1·16–1·52)	p<0·001	1·46 (1·32–1·61)	p<0·001
Adjusted for ever self-harmed by 15 years (n=2351)	1·30 (1·14–1·49)	p<0·001	1·33 (1·19–1·48)	p<0·001
Additionally adjusted for all covariates* (n=2351)	1·28 (1·11–1·47)	p<0·001	1·33 (1·19–1·48)	p<0·001
Imputed confounders (n=3694)	1·20 (1·08–1·33)	p<0·001 FMI 0·155	1·28 (1·18–1·39)	p<0·001 FMI† 0·155
Imputed outcome (n=5342)	1·18 (1·06–1·31)	p=0·002 FMI 0·472	1·25 (1·16–1·36)	p<0·001 FMI 0·469

OR=odds ratio. FMI=fraction of missing information. sMFQ=Short Mood and Feelings Questionnaire.

* Gender, previous depression (an sMFQ score of >11 at ages 10, 13, or 16 years), internalising and externalising difficulties at 11 years, self-perception, victimisation by bullies, antenatal depression, maternal history of depression, maternal education, and temperament (emotionality and activity).

† The FMI can be used to judge whether the number of imputations is sufficient for analysis.^32^ A rule of thumb is that the number of imputations (M) should be more than or equal to 100 × FMI. In our case, the number of imputations (M=100) was sufficient.

## Supplementary Material

Supplementary appendix

Supplementary audio Lucy Bowes and Niall Boyce discuss the association between goth subculture identification, depression, and self-harm

## References

[1] Gore FM, Bloem PJ, Patton GC (2011). Global burden of disease in young people aged 10–24 years: a systematic analysis. Lancet.

[2] Green H, McGinnity A, Meltzer H (2005). Mental health of children and young people in Great Britain, 2004.

[3] Thapar A, Collishaw S, Pine DS (2012). Depression in adolescence. Lancet.

[4] Prinstein M, Aikins J (2004). Cognitive moderators of the longitudinal association between peer rejection and adolescent depressive symptoms. J Abnorm Child Psychol.

[5] Hogue A, Steinberg L (1995). Homophily of internalized distress in adolescent peer groups. Dev Psychol.

[6] Kiuru N, Burk WJ, Laursen B (2012). Is depression contagious? A test of alternative peer socialization mechanisms of depressive symptoms in adolescent peer networks. J Adolesc Health.

[7] Conway CC, Rancourt D, Adelman CB (2011). Depression socialization within friendship groups at the transition to adolescence: the roles of gender and group centrality as moderators of peer influence. J Abnorm Psychol.

[8] Joiner TE, Katz J (1999). Contagion of depressive symptoms and mood: meta-analytic review and explanations from cognitive, behavioral, and interpersonal viewpoints. Clin Psychol Sci Pract.

[9] Prinstein MJ, Heilbron N, Guerry JD (2010). Peer influence and nonsuicidal self injury: longitudinal results in community and clinically-referred adolescent samples. J Abnorm Child Psychol.

[10] Hawton K, Saunders KE, O'Connor RC (2012). Self-harm and suicide in adolescents. Lancet.

[11] Young R, Sweeting H, West P (2006). Prevalence of deliberate self harm and attempted suicide within contemporary Goth youth subculture: longitudinal cohort study. BMJ.

[12] Rutledge CM, Rimer D, Scott M (2008). Vulnerable goth teens: the role of schools in this psychosocial high-risk culture. J Sch Health.

[13] Boyd A, Golding J, Macleod J (2013). Cohort profile: the ‘children of the 90s’—the index offspring of the Avon Longitudinal Study of Parents and Children. Int J Epidemiol.

[14] Mosbach P, Leventhal H (1988). Peer group identification and smoking: implications for intervention. J Abnorm Psychol.

[15] La Greca AM, Prinstein MJ, Fetter MD (2001). Adolescent peer crowd affiliation: linkages with health-risk behaviors and close friendships. Journal Pediatr Psychol.

[16] Goodman A, Heiervang E, Collishaw S (2011). The ‘DAWBA bands’ as an ordered-categorical measure of child mental health: description and validation in British and Norwegian samples. Soc Psychiatry Psychiatr Epidemiol.

[17] Lewis G (1994). Assessing psychiatric disorder with a human interviewer or a computer. J Epidemiol Community Health.

[18] Bebbington P, Dunn G, Jenkins R (2003). The influence of age and sex on the prevalence of depressive conditions: report from the National Survey of Psychiatric Morbidity. Int Rev Psychiatry.

[19] Office of Population Censuses and Surveys (1990). Standard occupational classification.

[20] Cox JL, Holden JM, Sagovsky R (1987). Detection of postnatal depression. Development of the 10-item Edinburgh postnatal depression scale. Br J Psychiatry.

[21] Wolke D, Woods S, Bloomfield L (2001). Bullying involvement in primary school and common health problems. Arch Dis Child.

[22] Barnett BE, Hanna B, Parker G (1983). Life event scales for obstetric groups. J Psychosom Res.

[23] Brown GW, Harris TO (1989). Life events and illness.

[24] Goodman R (2001). Psychometric properties of the Strengths and Difficulties Questionnaire. J Am Acad Child Adolesc Psychiatry.

[25] Angold A, Costello EJ, Messer SC (1995). The development of a short questionnaire for use in epidemiological studies of depression in children and adolescents. Int J Method Psych Psychiatr Res.

[26] Turner N, Joinson C, Peters TJ (2014). Validity of the short mood and feelings questionnaire in late adolescence. Psychol Assess.

[27] Goodman R, Ford T, Richards H (2000). The development and well-being assessment: description and initial validation of an integrated assessment of child and adolescent psychopathology. J Child Psychol Psychiatry.

[28] Harter S (1985). Manual for the self-perception profile for children.

[29] Buss AH, Plomin R. Temperament: early developing personality traits, 1984.

[30] Young R, Sweeting H, West P (2006). Self harm in Goth youth subculture: authors' reply. BMJ.

[31] Royston P (2005). Multiple imputation of missing values: update. Stata J.

[32] White I, Royston P, Wood AM (2011). Multiple imputation using chained equations: issues and guidance for practice. Stat Med.

[33] Little RJA, Rubin DB (2002). Statistical analysis with missing data.

[34] Kapur N, Cooper J, O'Connor RC (2013). Non-suicidal self-injury v. attempted suicide: new diagnosis or false dichotomy?. Br J Psychiatry.

[35] Muehlenkamp JJ, Kerr PL (2010). Untangling a complex web: how non-suicidal self-injury and suicide attempts differ. Prev Res.

[36] Nock MK (2010). Self-injury. Annu Rev Clin Psychol.

[37] Stanley B, Winchel R, Molcho A (1992). Suicide and the self-harm continuum: phenomenological and biochemical evidence. Int Rev Psychiatry.

[38] Wilkinson P (2013). Non-suicidal self-injury. Eur Child Adolesc Psychiatry.

[39] Young R, Sproeber N, Groschwitz RC (2014). Why alternative teenagers self-harm: exploring the link between non-suicidal self-injury, attempted suicide and adolescent identity. BMC Psychiatry.

[40] Rosen PM, Walsh BW (1989). Patterns of contagion in self-mutilation epidemics. Am J Psychiatry.

[41] Stevens EA, Prinstein MJ (2005). Peer contagion of depressogenic attributional styles among adolescents: a longitudinal study. J Abnorm Child Psychol.

