# Supplementary material for: Risk of depression and self-harm in teenagers identifying with goth subculture: a longitudinal cohort study
Source: Lancet Psychiatry. 2015 Aug 27;2(9):793–800. doi: 10.1016/S2215-0366(15)00164-9 (PMC4698805; doi:10.1016/S2215-0366(15)00164-9)
Supplement: Supplementary appendix [file mmc1.pdf]

THE LANCET Psychiatry

Supplementary appendix

This appendix formed part of the original submission and has been peer reviewed. We post it as supplied by the authors.

Supplement to: Bowes L, Carnegie R, Pearson R, et al. Risk of depression and self-harm in teenagers identifying with goth subculture: a longitudinal cohort study. *Lancet Psychiatry* 2015; published online Aug 28. http://dx.doi.org/10.1016/S2215-0366(15)00164-9.

Variables used to construct an imputation model for missing data analyses.

Variable	Age at assessment	Measure	Informant
Gender	Birth	-	Maternal report
Goth identification	15 years	Adapted from the Peer Crowd Questionnaire[1,2]	Self-report
Depression	15 years<br>18 years	DAWBA[3]<br>CIS-R[4]	Self-report
Self-harm/ suicidal intent	15 years<br>18 years	DAWBA[3]<br>CIS-R[4]	Self-report
Depressive symptoms	10, 13, 16 years	Short Mood and Feelings Questionnaire[5]	Self-report
Internalizing symptoms	11 years	Strengths and Difficulties Questionnaire[5]	Maternal report
Externalizing symptoms	11 years	Strengths and Difficulties Questionnaire[5]	Maternal report
Child temperament: emotionality/ activity/ shyness and sociability	6 years	Emotionality, Activity, and Sociability Temperament Scale[6]	Maternal report
Maternal depression in pregnancy	18 weeks gestation	Edinburgh Postnatal Depression Scale[7]	Maternal report
Maternal history of depression	12 weeks gestation	Single item (past history of severe depression yes/no)	Maternal report
Maternal depressive symptoms	18 weeks and 32 weeks gestation, 8 weeks, 8, 21 & 33 months, 5 years, 6 year	Edinburgh Postnatal Depression Scale[7]	Maternal report
Maternal education	Pregnancy	Percentage of mothers with a university degree versus without a degree	Maternal report
Paternal education	Pregnancy	Percentage of fathers with a university degree versus without a degree	Maternal report
Parental social class	Pregnancy	Lower of mother and father occupational social class, dichotomised into professional, managerial, or skilled professions versus partly or unskilled occupations[8]	Maternal report
Cannabis use	16 years	Cannabis Abuse Screen Test (CAST) [9]	Self-report
Peer victimization	8 & 10 years	Modified version of the Bullying and Friendship Interview Schedule[10]	Self-report
Peer bullying	8, 10 and 13 years	Modified version of the Bullying and Friendship Interview Schedule[10]	Self-report
Early death in family	5 years	Item from Life Events Questionnaire[11, 12]	Maternal report
Self esteem- global self worth score and scholastic competence score	8 years	Shortened form of Harter's Self Perception Profile for Children[13]	Self-report
Body image	13 years		Self-report

References

1 Mosbach P, Leventhal H. Peer group identification and smoking: implications for intervention. J Abnorm Psychol 1988; 97: 238–45.

2 La Greca AM, Prinstein MJ, Fetter MD. Adolescent peer crowd affiliation: linkages with health-risk behaviors and close friendships. J Pediatr Psychol 2001; 26: 131–43.

3 Goodman R, Ford T, Richards H, et al. The development and well-being assessment: description and initial validation of an integrated assessment of child and adolescent psychopathology. J Child Psychol Psych 2000; 41: 645–55.

4 Lewis G. Assessing psychiatric disorder with a human interviewer or a computer. J Epidemiol Comm Health 1994; 48: 207–10.

5 Angold A, Costello EJ, Messer SC, et al. The development of a short questionnaire for use in

epidemiological studies of depression in children and adolescents. Int J Method Psych 1995; 5: 237–49.
[6]Buss AH, Plomin R. Temperament: Early developing personality traits, 1984
[7] Cox JL, Holden JM, Sagovsky R. Detection of postnatal depression. Development of the 10-item Edinburgh postnatal depression scale. Br J Psychiatry 1987; 150: 782–86.
[8] Office of Population Censuses and Surveys. Standard occupational classification. London: HMSO, 1990.
[9] Legleye S, Karila L, Beck F, Reynaud M. Validation of the CAST, a general population Cannabis Abuse Screening Test. J. Subst. Use 2007; 12: 233–242.
[10]Wolke D, Woods S, Bloomfield L, et al. Bullying involvement in primary school and common health problems. Arch Dis Child 2001; 85: 197–201.
[11] Barnett BE, Hanna B, Parker G. Life event scales for obstetric groups. J Psychosom Res 1983; 27: 313 - 20.
[12]Brown GW, Harris TO. Life events and illness. New York: Guilford Press, 1989.
[13] Harter S. Manual for the self-perception profile for children. Denver: University of Denver, 1985.
